# MiR-26 enhances chemosensitivity and promotes apoptosis of hepatocellular carcinoma cells through inhibiting autophagy

**DOI:** 10.1038/cddis.2016.461

**Published:** 2017-01-12

**Authors:** Fangfang Jin, Yanbo Wang, Mingzhen Li, Yanan Zhu, Hongwei Liang, Chen Wang, Feng Wang, Chen-Yu Zhang, Ke Zen, Limin Li

**Affiliations:** 1 State Key Laboratory of Pharmaceutical Biotechnology, Nanjing University Advanced Institute of Life Sciences, Jiangsu Engineering Research Center for MicroRNA Biology and Biotechnology, Nanjing University, Nanjing, Jiangsu, China; 2 Affiliated Gulou Hospital, Medical College of Nanjing University, Nanjing, Jiangsu, China

## Abstract

Hepatocellular carcinoma (HCC) generally possesses a high resistance to chemotherapy. Given that autophagy is an important factor promoting tumor chemoresistance and HCC express low level of miR-26, we aim to investigate the functional role of miR-26 in autophagy-mediated chemoresistance of HCC. We found that chemotherapeutic drug doxorubicin (Dox) induced autophagy but decreased the level of miR-26a/b in HCC cells. Activating autophagy using rapamycin can directly downregulate the level of miR-26a/b in HCC cells. In turn, restoring the expression of miR-26a/b inhibited autophagy induced by Dox and promoted apoptosis in HCC cells. Further mechanistic study identified that miR-26a and miR-26b target ULK1, a critical initiator of autophagy, at post-transcriptional level. Results from 30 cases of patients with HCC also showed that tumor cellular levels of miR-26a and miR-26b are significantly downregulated as compared with the corresponding control tissues and negatively correlated with the protein level of ULK1 but are not correlated to the mRNA level of ULK1. Gain- and loss-of-function assay confirmed that miR-26a/b inhibited autophagic flux at the initial stage through targeting ULK1. Overexpression of miR-26a/b enhanced the sensitivity of HCC cells to Dox and promoted apoptosis via inhibiting autophagy *in vitro*. Using xenograft models in nude mice, we confirmed that miR-26a/b, via inhibiting autophagy, promoted apoptosis and sensitized hepatomas to Dox treatment *in vivo*. Our findings demonstrate for the first time that miR-26a/b can promote apoptosis and sensitize HCC to chemotherapy via suppressing the expression of autophagy initiator ULK1, and provide the reduction of miR-26a/b in HCC as a novel mechanism of tumor chemoresistance.

Hepatocellular carcinoma (HCC) is the fifth most common malignancy globally and the third most common cause of cancer-related death.^1^ Surgery is the main approach used to treat this disease; however, because few patients have the opportunity to avail of surgery, other treatments such as chemotherapy are widely used, especially for advanced-stage HCC.^2^ Approximately 70% of patients with HCC have an extremely poor prognosis because of high recurrence and the lack of treatment modalities.^1^ One of the main reasons for this failure may be its chemoresistance.^3^ Therefore, understanding the molecular mechanisms involved in the chemoresistance of HCC may lead to improved clinical outcomes. Among the many factors,^3^ therapy-induced autophagy represents a novel mechanism of resistance to anticancer therapy.^4^

Autophagy is an evolutionarily conserved process by which damaged or excessive organelles and cytoplasmic proteins are degraded through lysosomal degradation.^5^ It has been implicated in a variety of human diseases.^6, 7^ For example, autophagy often contributes to tumor chemotherapy resistance and cancer cell survival under various stresses.^8, 9, 10^ Recently, autophagy inhibitors have been used to enhance the sensitivity of various cancers toward chemotherapy.^11, 12^ For instance, the autophagy inhibitors, hydroxychloroquine (CQ) and 3-methyladenine (3-MA), have been used to treat advanced non-small-cell lung cancer^13^ and colorectal cancer cells.^12, 14^ Consequently, developing strategies to inhibit autophagy and sensitize HCC cells to metabolic stress may be promising for HCC chemotherapy.

MicroRNAs (miRNAs) can regulate diverse cellular functions at the post-transcriptional level and have important roles in a wide variety of physiological and pathological cellular processes.^15, 16, 17^ Recently, a series of miRNAs have been found to be involved in the modulation of autophagy. However, our understanding of the relationship between miRNAs and autophagy in HCC remains limited. Thus far, only some miRNAs, including miR-224, miR-100, miR-101, miR-199a and miR-375, have been confirmed to modulate HCC chemosensitivity through targeting autophagy.^8, 18, 19, 20, 21^ Little is known about how miRNAs can be used to turn off autophagy at the initiating stage. Therefore, it is very important to develop strategies to inhibit autophagy initiation and restore drug sensitivity in HCC.^22^ Recent studies have indicated that miRNA-26 is downregulated in HCC, breast cancer, anaplastic thyroid cancer and nasopharyngeal cancer.^23, 24, 25^ A previous study showed that the therapeutic delivery of miR-26a by an adeno-associated virus can inhibit HCC cell proliferation and induce tumor-specific apoptosis.^26^ Moreover, HCC patients with poor miR-26a expression exhibited shorter overall survival.^27^ However, the mechanism by which miR-26 acts in HCC therapy remains unclear. Thus, we proposed that whether miR-26 affects the chemotherapy of HCC by targeting autophagy.

In this study, we found for the first time that members of the miR-26 family (miR-26a and miR-26b, miR-26a/b) can act as potential autophagy inhibitors to sensitize HCC cells to doxorubicin (Dox) and promotes apoptosis by directly inhibiting the expression of serine/threonine protein kinase ULK1, a critical initiator of autophagy. Our study not only illustrated the role of miR-26a/b as novel modulators of autophagy at an early stage by targeting ULK1 but also demonstrated that miR-26a/b can be used as autophagy inhibitors to increase the chemosensitivity of HCC and promotes apoptosis *in vitro* and *in vivo*. These results provide new insight into the role of miR-26a/b dysregulation in the autophagy-mediated chemoresistance of HCC and imply a strategy that can enhance HCC chemosensitivity using miRNAs.

## Results

### Dox-induced autophagy decreases the level of miR-26a/b, and miR-26a/b promotes apoptosis and inhibits viability of HCC cells through downregulating autophagy

**Figure 1:**
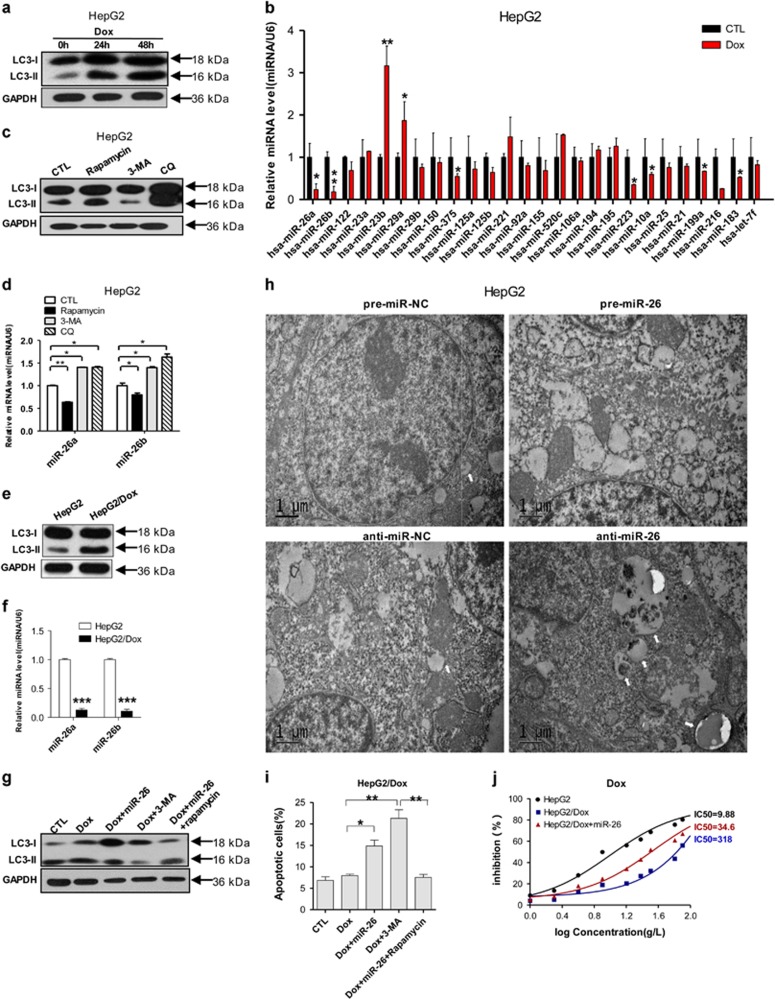
Dox-induced autophagy decreases miR-26a/b levels, and miR-26a/b promotes apoptosis and inhibits viability and proliferation of HCC cells through downregulating autophagy. (**a**) Representative western blotting analyses of LC3-I and LC3-II in HepG2 cells after treatment with Dox for 24 and 48 h. (**b**) Relative levels of miRNAs in HepG2 cells after treatment with Dox for 48 h were analyzed using RT-qPCR. (**c**) Representative western blotting analyses of LC3-I and LC3-II in HepG2 cells after treatment with or without rapamycin, 3-MA and CQ for 24 h. (**d**) miR-26a/b were detected using RT-qPCR in HepG2 cells after treatment with/without 3-MA, CQ and Rapamycin for 24 h. (**e**) Protein levels of LC3-I and LC3-II in HepG2 and HepG2/Dox cells. (**f**) Relative miR-26a/b levels in HepG2, HepG2/Dox cells. (**g**) Representative western blotting analyses of LC3-I and LC3-II in HepG2/Dox cells transfected with/without miR-26a/b mimics under different treatments for 24 h. (**h**) The ultrastructure of treated HepG2 cells were observed by electron micrography after 24-h transfection. White arrows showed autophagosomes or autophagosome-fused lysosomes. (**i**) Apoptosis of HepG2/Dox cells transfected with or without miR-26a/b mimics under various treatments were analyzed. (**j**) The sensitivities of HepG2 and HepG2/Dox cells under different treatments were determined using a CCK-8 assay. Statistical data are presented as the means±S.E. from three independent experiments. *P<0.05; **P<0.01; and ***P<0.001

Dox has been widely used for clinical chemotherapy in patients with HCC.^28^ In this study, autophagy was induced by Dox in HCC cells. As shown in Figure 1a, Dox treatment significantly induced the progression of LC3-I to LC3-II over time. To investigate the expression profiles of miRNAs under Dox treatment, we evaluated the levels of 26 miRNAs with abnormal expression in liver-related diseases. RT-qPCR results showed that there are nine miRNAs with considerable change of expression levels after Dox treatment, among which miR-26a/b are the most significantly downregulated miRNAs (Figure 1b). To ascertain whether autophagy can lead to the downregulation of miR-26a/b, we introduced 3-MA, lysosome–autophagosome fusion inhibitor CQ and rapamycin into HepG2 cells to modulate autophagy. As expected, 3-MA and CQ increased the levels of miR-26a/b, whereas rapamycin decreased the levels of miR-26a/b, indicating that autophagy can promote the degradation of miR-26a/b in HepG2 cells (Figure 1d).

To figure out the relationship between miR-26a/b and autophagy, we established Dox-resistant HepG2 cells (HepG2/Dox), and found that HepG2/Dox cells showed higher LC3-II level (Figure 1e) and lower miR-26a/b levels (Figure 1f). Furthermore, as the same to 3-MA treatment, inhibition autophagy through miR-26a/b overexpression promoted apoptosis of HepG2/Dox cells, and rapamycin treatment inhibited the promotion effect of miR-26a/b overexpression on Dox-induced apoptosis (Figures 1g–i, Supplementary Figure 2). These data indicate that miR-26a/b promoted apoptosis through targeting autophagy at early stage. TEM results showed that autophagosomes and autophagosome-fused lysosomes could be reduced in pre-miR-26a/b transfected HepG2 cells and neutralization of miR-26a/b with their inhibitors restored the activation of autophagy. These data support that the decrease of LC3-II by miR-26a/b resulted from the inhibition of autophagosome formation and not from excessive autophagosome degradation (Figure 1h). Furthermore, the IC50 values of miR-26a/b-overexpressing HepG2/Dox cells were much lower than that of HepG2/Dox cells without miR-26a/b overexpression (Figure 1j). Thus, these results suggest that Dox induce autophagy and decrease the expression of miR-26a/b in HepG2 cells, and in turn, miR-26a/b promotes the apoptosis and inhibit the proliferation and viability of HCC cells through targeting autophagy.

### MiR-26a/b inhibits autophagy at an early stage by directly targeting ULK1 in HCC cells

**Figure 2:**
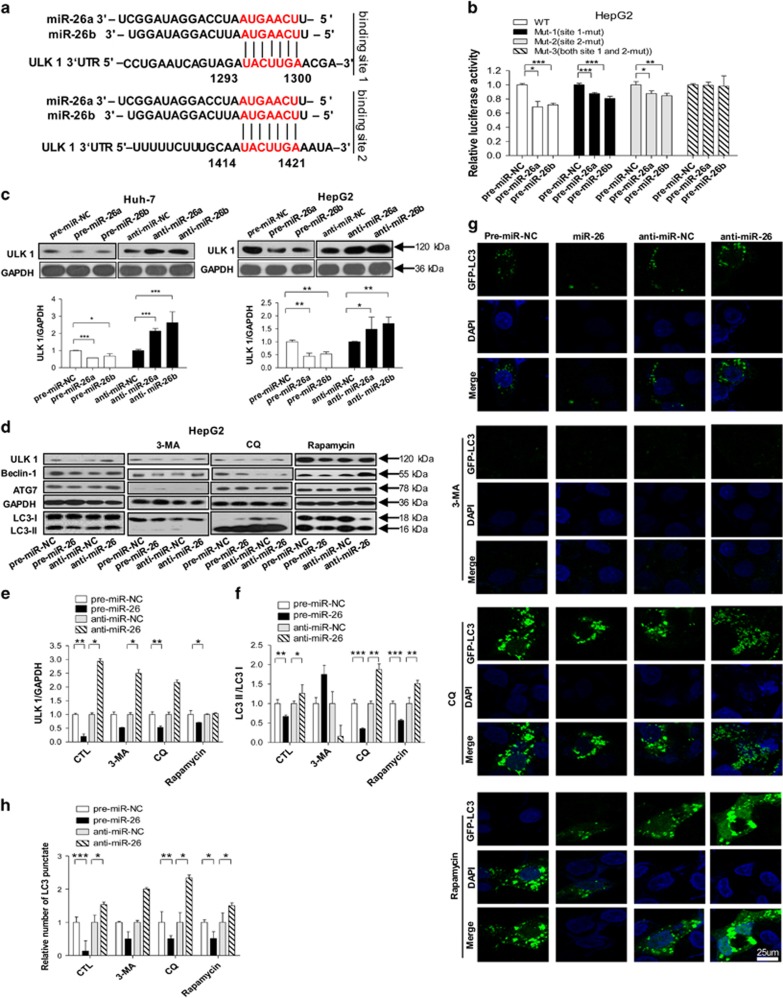
MiR-26a/b directly targets ULK1 and inhibits autophagy at the initial stage in HCC cells. (**a**) A schematic diagram of ULK1 3′-UTR as a putative target for miR-26a/b. The seed-recognizing sites in the ULK1 3′-UTR by miR-26a/b are indicated in red. (**b**) Relative luciferase activity in 293T that were transfected with firefly luciferase reporters containing WT or mutant 3′-UTRs of ULK1, pre-miR-26a/b or random pre-miR-NC. (**c**) Huh-7 and HepG2 cells were transfected with pre-miR-NC, pre-miR-26a/b or anti-miR-NC, anti-miR-26a/b. The ULK1 expression level were detected using immunoblotting. (**d**) HepG2 cells were transfected with pre-miR-NC, pre-miR-26 or anti-miR-26 for 24 h, cells were treated with/without 3-MA, CQ or rapamycin for 24 h. The expression levels of LC3, ULK1, Beclin-1 and ATG7 were detected. (**e**) Quantitative analysis of ULK1 protein levels. (**f**) Quantitative analysis of LC3-II/LC3-I protein levels. (**g**) HepG2 cells were transfected with GFP-LC3 plasmid, pre-miRNA-NC, miR-26a/b mimics, anti-miR-NC or anti-miR-26; after 24-h transfection, cells were treated with 3-MA, CQ or rapamycin for a further 24 h before observation to count GFP-LC3 puncta under confocal microscopy. Blue indicates DAPI-stained nuclei. Green indicates GFP-LC3. One of 10 representative micrographs is shown. (**h**) The relative number of GFP-LC3 punctae in cells treated with 3-MA, CQ or rapamycin was calculated from 10 random fields. The data are presented as the means±S.E. obtained from three independent experiments. *P<0.05; **P<0.01; and ***P<0.001

To explore the mechanisms underlying the above phenomena, the candidate targets of miR-26a/b were predicted using a combination of three databases: TargetScan, miRanda and PicTar. ULK1, an essential gene at the initiating stage of autophagy,^29^ was consistently predicted by three servers, and 3′-untranslated region (UTR) of ULK1 contained two conserved binding sites for miR-26a/b. The predicted interactions between miR-26a/b and targeting sites within the 3′-UTR of ULK1 are illustrated in Figure 2a. To validate the binding of miR-26a/b to ULK1 3′-UTR, the full-length 3′-UTR of ULK1 mRNA was amplified and fused downstream of the firefly luciferase gene in a reporter plasmid. The constructed plasmids were transfected into human 293T cells together with control plasmid (*β*-gal) and miR-26a/b mimics. Luciferase reporter assays demonstrated that miR-26a/b overexpression suppressed approximately 30% of the luciferase activity of the reporters compared with the cells transfected with control mimics (Supplementary Figure 1, Figure 2b). For further validation, point mutations were introduced into the sites that are complementary to miR-26a/b within ULK1 mRNA 3′-UTR. As shown in Figure 2b, when either of the two binding sites was mutated, the luciferase activity was still reduced; when both binding sites were mutated, miR-26a/b overexpression caused a loss of luciferase inhibition, indicating that miR-26a/b directly recognize both predicted sites in 3′-UTR of the ULK1 mRNA.

We then assessed the protein and mRNA levels of ULK1 in HepG2 and Huh-7 cells after transfecting with miR-26a/b mimics and inhibitors. As expected, miR-26a/b overexpression or knockdown significantly reduced or increased the level of ULK1 protein in HCC cells (Supplementary Figures 2A and C, Figure 2c), but did not affect ULK1 mRNA levels (Supplementary Figures 2B and D). At the same time, we examined the effect of miR-26a/b overexpression or knockdown on the proteins downstream of ULK1 that are involved in autophagy. As shown in Figures 2d and h, the overexpression of miR-26a/b significantly decreased the expression of ULK1, Beclin-1 and ATG7, the transfer of LC3-I to LC3-II (Figures 2d–f), and the number of cells with punctate GFP-LC3 (Figures 2g and h); however, depletion of miR-26a/b increased the expression of ULK1, Beclin-1 and ATG7, the transfer of LC3-I to LC3-II (Figures 2d–f), and cells with punctate GFP-LC3 (Figures 2g and h), compared with cells that were transfected with control oligonucleotides. To demonstrate that miR-26a/b control autophagy at the initiating stage, rapamycin, 3-MA and the lysosome–autophagosome fusion inhibitor CQ were added to HepG2 cells after miR-26a/b administration. The results showed that 3-MA administration strongly inhibited the transfer of LC3-I to LC3-II (Figures 2d and f) and decreased the number of cells with punctate GFP-LC3 (Figures 2g and h) but did not affect the effect of miR-26a/b on regulating the protein expression of ULK1 and Beclin-1 (Figures 2d and e). Adding CQ significantly promoted LC3-II accumulation (Figures 2d and f) and increased the number of cells with punctate GFP-LC3 (Figures 2g and h) but did not affect the regulatory effect of miR-26a/b on ULK1, Beclin-1 and ATG7, and the transfer of LC3-I to LC3-II (Figures 2d–f). Moreover, treatment with rapamycin significantly promoted the regulatory effect of miR-26a/b on the protein expression of ULK1, Beclin-1 and ATG7, as well as the transfer of LC3-I to LC3-II (Figures 2d–f) and number of cells with punctate GFP-LC3 (Figures 2g and h). Taken together, these results suggest that miR-26a/b directly suppress post-transcriptional ULK1 expression and inhibit autophagy at an early stage.

**Figure 3:**
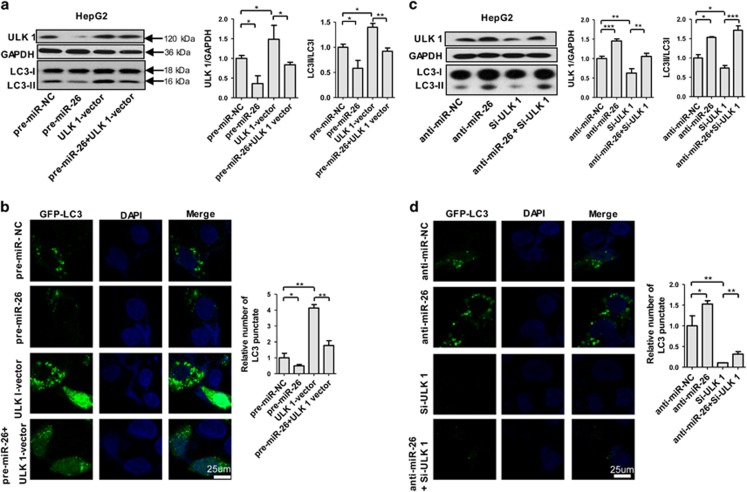
MiR-26a/b regulates autophagy through targeting ULK1. (**a**) Protein levels of ULK1 and LC3 were determined in HepG2 cells overexpressed with pre-miR-NC, pre-miR-26, ULK1-vector or pre-miR-26 plus ULK1-expressing plasmids. GAPDH was served as internal control. The right histograms represent quantitative analysis of ULK1 and LC3-II/LC3-I protein level. (**b**) Representative photographs of HepG2 cells transfected with GFP-LC3-expressing plasmids plus pre-miR-NC, pre-miR-26, ULK1-vector or pre-miR-26+ULK1-vector. (**c**) Protein levels of ULK1 and LC3 were detected in HepG2 cells overexpressed with anti-miR-NC, anti-miR-26, Si-ULK1 or anti-miR-26 plus Si-ULK1. (**d**) Representative photographs of HepG2 cells transfected with GFP-LC3-expressing plasmids plus anti-miR-NC, anti-miR-26, Si-ULK1 or anti-miR-26+Si-ULK1. The right histogram represents quantitative analysis of GFP-LC3 punctate from 10 micrographs. *P<0.05; **P<0.01; and ***P<0.001

To examine further whether the regulatory role of miR-26 in autophagy is dependent on ULK1, we constructed ULK1-expressing plasmids and siRNA to evaluate whether co-transfected miR-26 mimics or inhibitors counteract the overexpression or knockdown effect of ULK1-expressing plasmids or ULK1 siRNA in HepG2 cells, respectively. The results showed that ULK1 overexpression significantly increased ULK1 expression (Supplementary Figure 4, Figure 3a), the transfer of LC3-I to LC3-II (Figure 3a) and number of cells with punctate GFP-LC3 (Figure 3b). However, overexpression of miR-26a/b in ULK1-overexpressing cells rescued ULK1 level (Figure 3a), the transfer of LC3-I to LC3-II (Figure 3a) and number of cells with punctate GFP-LC3 (Figure 3b). Conversely, ULK1 knockdown decreased ULK1 expression (Supplementary Figure 3, Figure 3c), transfer of LC3-I to LC3-II (Figure 3c) and number of cells with punctate GFP-LC3 (Figure 3d). When ULK1 siRNA was co-transfected with miR-26 inhibitor, the decreases in ULK1 expression (Supplementary Figure 3), transfer of LC3-I to LC3-II (Figure 3c) and number of cells with punctate GFP-LC3 (Figure 3d) were abolished. Taken together, these data demonstrate that miR-26 functions as an early suppressor of autophagy in HCC by targeting ULK1.

### MiR-26a/b is inversely correlated with the protein level of ULK1 and increased autophagy in tumor tissues of patients with HCC

**Figure 4:**
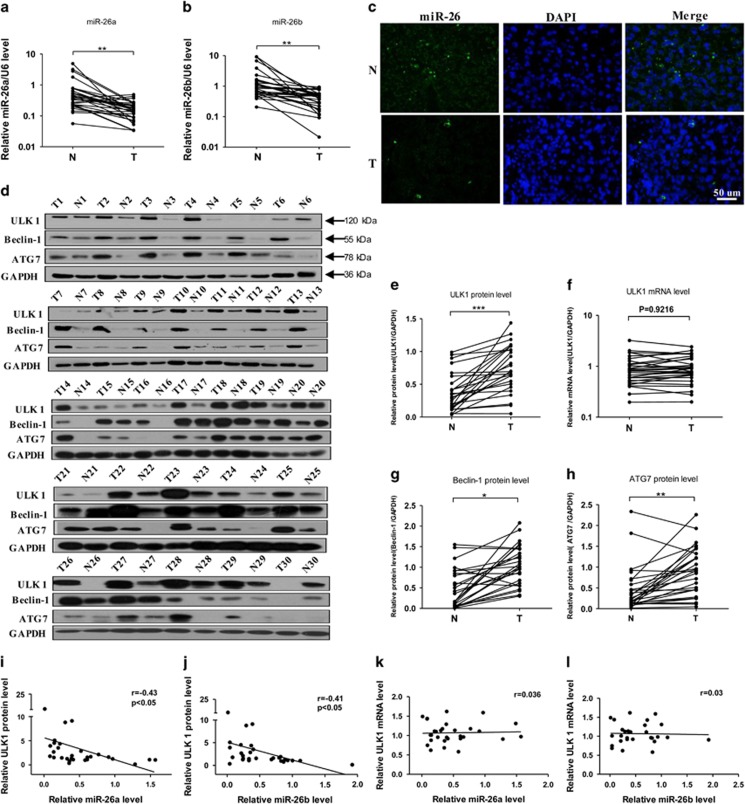
MiR-26a/b is inversely correlated with increased autophagy in tumor tissues of patients with HCC. (**a**–**c**) Relative levels of miR-26a/b (expressed as the miRNA/U6 ratio) in tumor tissues (T) and the corresponding background livers (N) were determined using RT-qPCR and *in situ* hybridization assays. Data are shown as the means±S.E.M.; *n*=30 in each group. (**d**) Protein levels of ULK1, Beclin-1 and ATG7 were determined using western blotting in 30 pairs of samples. GAPDH was used as an internal control. (**e**–**h**) Quantitative analyses of the protein and mRNA levels of ULK1, Beclin-1 and ATG7 in 30 pairs of HCC samples. (**i**–**l**) Pearson's correlation scatter plot of the fold changes of miR-26a/b and ULK1 protein, mRNA in HCC tissues. *P<0.05; **P<0.01; and ***P<0.001

Next, we determined whether miR-26a/b are correlated with ULK1 and autophagy in HCC patients. For this experiment, tumor tissue and background liver tissue obtained from 30 patients with HCC (Table 1) were analyzed. Compared with the corresponding control tissues, miR-26a/b (Figures 4a–c) were significantly downregulated in 27/28 HCC cases respectively. In contrast, protein levels of ULK1 were significantly upregulated in 27 HCC cases (Figures 4d and e), though the mRNA levels of ULK1 did not differ significantly (Figure 4f). Moreover, Beclin-1 and ATG7, which act downstream of ULK1, were also significantly upregulated in tumor tissues compared with the corresponding background tissues (Figures 4g and h). To further clarify the relationship between miR-26a/b and ULK1, we performed a correlation analysis between miR-26a/b and ULK1. According to the results, the expression levels of miR-26a/b (Figures 4i and j) are significantly and negatively correlated with ULK1 protein level but are not correlated to ULK1 mRNA level (Figures 4k and l). This finding is consistent with our previous results showing that miR-26a/b post-transcriptionally regulate the expression of ULK1 in HCC cells.

### MiR-26a/b enhances the sensitivity of HCC to Dox and promotes HCC apoptosis by inhibiting autophagy *in vitro*

**Figure 5:**
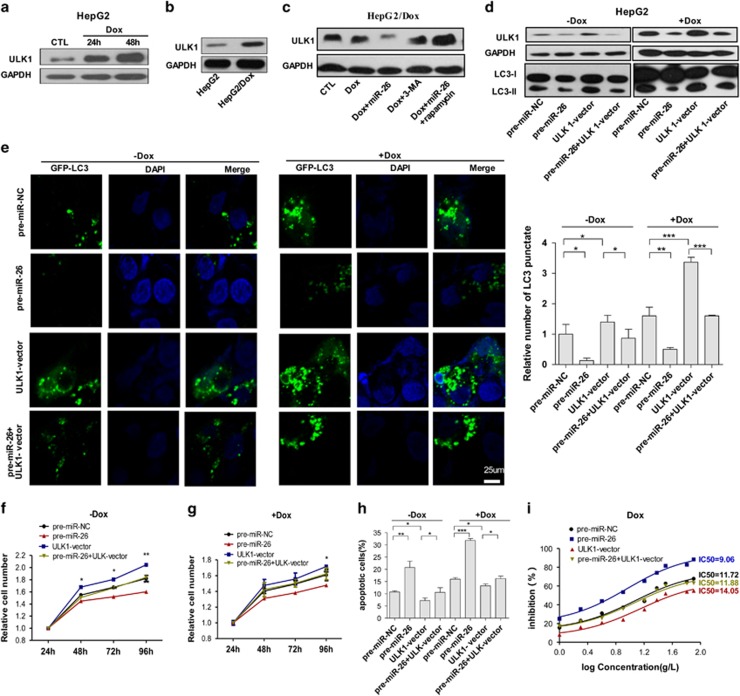
MiR-26a/b enhances the sensitivity of HCC cells to chemotherapeutic drugs and promotes HCC apoptosis by inhibiting autophagy *in vitro*. (**a**) Representative western blotting analyses of ULK1 in HepG2 cells after treatment with Dox over time. (**b**) Different expression levels of ULK1 in HepG2 and HepG2/Dox cells. (**c**) ULK1 levels in HepG2/Dox cells transfected with/without miR-26 mimics under different treatments. (**d**) HepG2 cells transfected with pre-miR-NC, pre-miR-26, ULK1-vector or pre-miR-26 plus ULK1-vector, treated with/without Dox. Protein levels of ULK1 and LC3 were determined. (**e**) Representative photographs of HepG2 cells transfected with GFP-LC3-expressing plasmids plus pre-miR-NC, pre-miR-26, ULK1-vector or pre-miR-26+ULK1-vector, treated with/without Dox. The right-hand histogram represents a quantitative analysis of GFP-LC3 punctae from 10 micrographs. (**f** and **g**) Cell viabilities of HepG2 cells under different treatments were determined using a CCK-8 assay at various time points. (**h**) Cell apoptosis of HepG2 cells under various treatments was analyzed using flow cytometry. (**i**) The sensitivities of HepG2 cells under different transfections with Dox were determined using a CCK-8 assay. *P<0.05; **P<0.01; and ***P<0.001

In view of the fact that drug-induced autophagy has an important role in drug resistance of HCC, we hypothesized that miR-26a/b may increase the sensitivity of HCC cells by attenuating the protective effect of autophagy through targeting ULK1. As shown in Figure 5a, Dox treatment significantly increased ULK1 protein level in HepG2 cells over time, and HepG2/Dox cells showed higher ULK1 level compared with HepG2 (Figure 5b). More importantly, as compared with 3-MA and Dox treatment, overexpressing miR-26a/b inhibited the ULK1 expression, and rapamycin treatment reversed the inhibitory effect of miR-26a/b on ULK1 in HepG2/Dox cells (Figure 5c). These data suggest that Dox induce autophagy at early stage. To demonstrate whether miR-26a/b inhibit autophagy through targeting ULK1, ULK1-expressing plasmids and miR-26a/b mimics were co-transfected into HCC cells with/without Dox treatment. The results showed that miR-26a/b decreases the ULK1 protein expression (Figure 5d), the progression of LC3-I to LC3-II (Figure 5d) and number of cells with punctate GFP-LC3 (Figure 5e) that are induced by Dox, whereas ULK1 overexpression increases ULK1 expression (Figure 5d), the transfer of LC3-I to LC3-II (Figure 5d) and number of cells with punctate GFP-LC3 (Figure 5e) that are induced by Dox. When HepG2 cells were co-transfected with miR-26a/b-expressing plasmids and ULK1-expressing plasmids, the effect of ULK1 on Dox treatment was largely abolished (Figures 5d and e). Furthermore, overexpression of miR-26a/b decreased cell viability (Figures 5e and f) and increased apoptosis (Figure 5g) in HCC cells with/without Dox treatment, whereas overexpression of ULK1 increased cell viability (Figures 5e and f) and decreased apoptosis (Figure 5g) in HCC cells with/without Dox treatment. Moreover, co-transfecting miR-26a/b-expressing plasmids with ULK1-vector abolished the upregulation of cell viability (Figures 5f and g) and the downregulation of apoptosis of HepG2 cells (Figure 5h) and Huh-7 cells (Supplementary Figure 5) that were induced by ULK1 in the presence/absence of Dox. We then assessed the potential effect of miR-26a/b on the sensitivity of HCC cells to Dox. The IC50 values of miR-26a/b-overexpressing HepG2 cells were much lower, and those of ULK1-overexpressing HepG2 cells were much higher, than the control (Figure 5i). Moreover, co-transfecting miR-26a/b with ULK1-expressing plasmids rescued and abolished the upregulatory effect of ULK1 on IC50 (Figure 5i and Supplementary Figure 6). Taken together, these results indicate that miR-26 can enhance the sensitivity of HCC cells to Dox by inhibiting autophagy and promoting cell apoptosis.

### MiR-26a/b inhibits autophagy and sensitizes hepatoma to Dox *in vivo*

**Figure 6:**
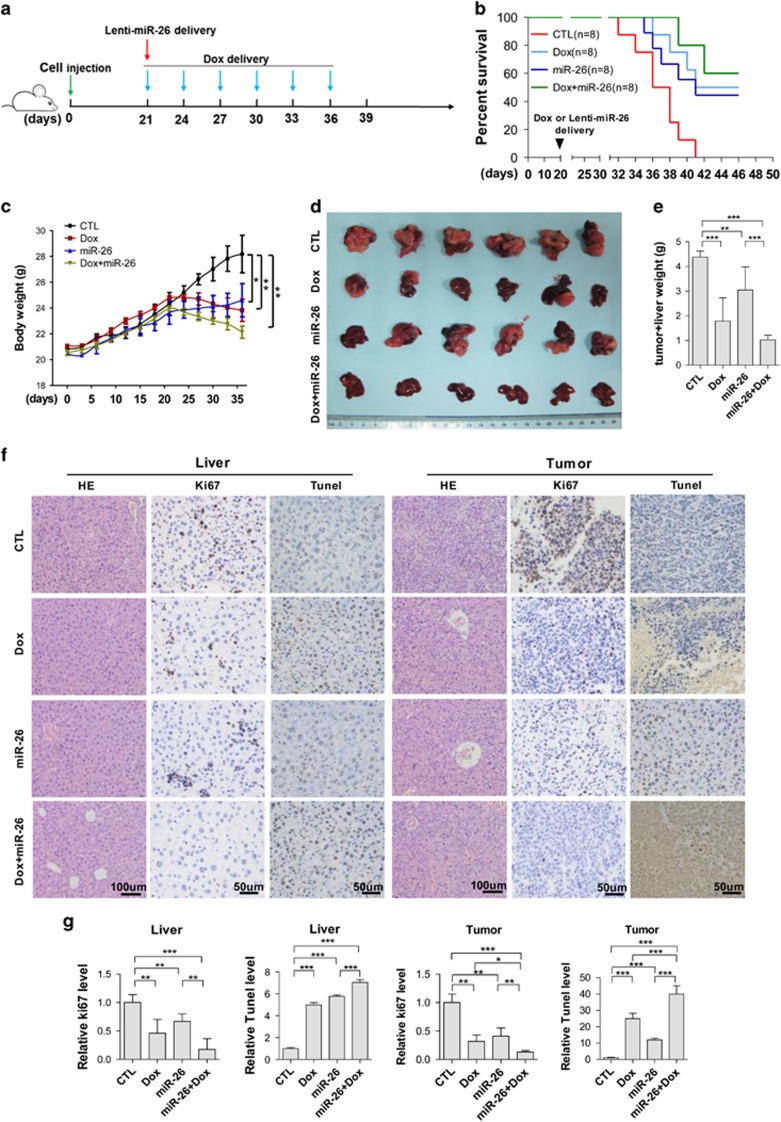
Intravenous injections of miR-26a/b-expressing lentivirus enhance the efficiency of chemotherapeutic drugs by blocking the growth of tumor *in vivo*. (**a**) A schematic diagram illustrating the experimental design. The HCC mouse model was constructed using HepG2 cells. Intravenous delivery of miR-26a/b-expressing lentivirus started at 3 weeks after orthotopic liver implantation in nude mice (day 0). Then, mice were administered with PBS or Dox every 3 days. Mice were divided into four groups according to the treatments: PBS (CTL), Lenti-miR-26 (miR-26), Dox and Dox plus Lenti-miR-26 (DOX+miR-26). (**b**) Survival analysis. (**c**)The time course of body weight. (**d**) Tumors at the week 5. (**e**) The quantitative analysis of tumor and liver weights. (**f**) HE, Ki67 and Tunel staining of tumor and liver sections obtained from the four mouse groups. (**g**) The histograms represent quantitative analyses of Ki67 and Tunel-positive signals in the tumor and liver sections. All data are shown as the means±S.E. obtained from three separate experiments. *P<0.05; **P<0.01; and ***P<0.001

Next, HepG2 cells stably expressing miR-26a/b were constructed using lentivirus-packaged miR-26a/b and lentivirus-packaged empty vector served as the control. The results showed that the stable expression of miR-26a/b in HepG2 cells inhibits the transfer of LC3-I to LC3-II (Supplementary Figure 7A). Moreover, HepG2 cells stably expressing miR-26a/b were more sensitive to the Dox-induced decrease of cell viability (Supplementary Figure 7B) and increase of cell apoptosis (Supplementary Figure 7C). Furthermore, compared with untreated HepG2 cells or HepG2 cells expressing lentivirus-packaged empty vector, HepG2 cells expressing miR-26a/b less easily formed tumors in the livers of nude mice (Supplementary Figure 7D). To further evaluate whether miR-26a/b improves the ability of chemotherapeutic drugs to block the growth of tumors by targeting autophagy *in vivo*, a mouse tumor model was developed using HepG2 cells. As shown in Figure 6a, 3 weeks after orthotopic liver implantation in nude mice, the mice were injected Dox or miR-26a/b-expressing lentiviruses through the tail vein. The mice were divided into four groups: PBS (CTL), Lenti-miR-26 (miR-26), Dox or Dox plus Lenti-miR-26 (DOX+miR-26). The results showed that the administration of miR-26 or Dox can significantly improve survival percentage (Figure 6b), decrease body weight (Figure 6c), tumor volume (Figure 6d) and liver plus tumor weight (Figure 6e) compared with PBS treatment. Importantly, when miR-26a/b was combined with Dox treatment, miR-26 further improved the therapeutic effect of Dox on tumor growth, including survival percentage (Figure 6b), body weight (Figure 6c), tumor volume (Figure 6d) and liver plus tumor weight (Figure 6e). Hematoxylin and eosin (H&E) staining of tumor/liver sections showed that the control group had a more invasive edge than the Dox, miR-26 and Dox plus miR-26 groups and that the Dox plus miR-26 group had a less invasive edge than the Dox and the miR-26 groups (Figure 6f). Ki67 and Tunel staining of the tumor/liver sections showed that the control group contained more Ki67-positive and less Tunel-positive cells than those of the Dox, miR-26 and Dox plus miR-26 groups and that Dox plus miR-26 group had fewer Ki67-positive and more Tunel-positive cells than the Dox and miR-26 groups (Figures 6f and g), respectively. Taken together, these results indicate that miR-26a/b can sensitize hepatomas to Dox *in vivo*.

**Figure 7:**
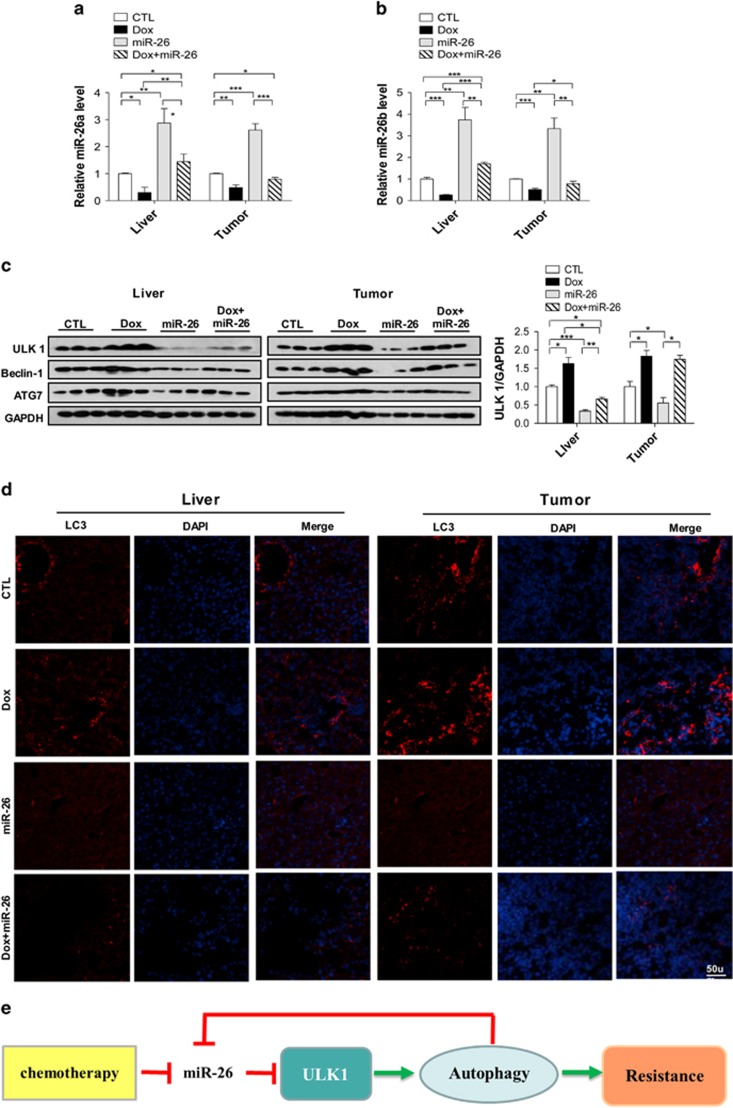
Intravenous injections of miR-26a/b-expressing lentivirus enhance the efficiency of chemotherapeutic drugs by inhibiting autophagy *in vivo*. (**a** and **b**) Quantitative analysis of miR-26a/b levels in tumors and livers of four mouse groups. (**c**) Western blotting analyses of ULK1, Beclin-1 and ATG7 proteins in the tumors and livers of mice that were treated with PBS, Lenti-miR-26, Dox or Dox plus Lenti-miR-26. (**d**) Representative micrographs of the immunofluorescence staining of LC3 puncta in tumor and liver tissues. Red indicates LC3; blue indicates nuclei. (**e**) Schematic illustrating how miR-26 induces chemosensitivity to drugs. MiR-26 inhibits autophagy and sensitize tumor cells to chemotherapy by suppressing ULK1 and downstream events. Pointed arrows and blunted arrows indicate activation and repression, respectively. *P<0.05; **P<0.01; and ***P<0.001

Next, we investigated whether the effect of miR-26a/b in sensitizing tumor cells to Dox occurred through inhibition of autophagy. The administration of miR-26a/b-expressing lentivirus or Dox significantly increased or decreased the level of miR-26a/b in the tumor, respectively, and infection of miR-26a/b-expressing lentivirus strongly reduced the downregulation of miR-26a/b that was induced by Dox (Figures 7a and b). Western blotting analysis showed that ULK1, Beclin-1 and ATG7 protein expression was much higher in tumors/livers obtained from the Dox group, whereas tissues from the miR-26 group exhibited much lower ULK1, Beclin-1 and ATG7 expression than control group; these results indicated that Dox treatment can induce autophagy, whereas miR-26a/b inhibits autophagy *in vivo* (Figure 7c). The combined use of miR-26-expressing lentivirus and Dox can strongly rescue the upregulatory effect of Dox on ULK1, Beclin-1 and ATG7 level (Figure 7c). Immunofluorescent staining of LC3 showed that tumors obtained from the Dox group exhibited much higher punctate LC3 signals, whereas miR-26 group exhibited much lower punctate LC3 signals than control group, indicating that Dox treatment can induce autophagic flux, and miR-26a/b inhibits autophagic flux, *in vivo* (Figure 7d). The combined utilization of miR-26-expressing lentivirus and Dox can strongly rescue the upregulatory effect of Dox on the accumulation of LC3 *in vivo* (Figure 7d). These results indicate that miR-26a/b can sensitize HCC to Dox treatment by inhibiting autophagy.

## Discussion

Under cellular stress conditions, such as nutrient deficiency, chemotherapy and radiation treatment, autophagy is rapidly activated to maintain the survival of tumor cells.^30, 31^ Therefore, autophagy has been proposed as a potential mechanism of cancer drug resistance. Accumulating evidence shows that modulating the level of autophagy may be useful as a therapeutic strategy to enhance the efficacy of many antitumor agents, including cisplatin, Dox and sorafenib.^4, 20, 32^ In our study, we found that the expression levels of miR-26a/b are decreased in Dox-treated HepG2 cells and implanted tumors. This decrease is likely due to the degradation of miR-26a/b by the induced autophagy. Infection of miR-26a/b-expressing lentiviruses in HepG2 cells can inhibit autophagy, sensitize cells to Dox-induced apoptosis. Previous reports ^27^ and the results obtained here have shown that miR-26a/b are generally downregulated in patients with HCC. In addition, miR-26 expression is also decreased in other tumors, such as bladder and breast cancer, oral squamous cell carcinoma, Burkitt lymphoma and rhabdomyosarcoma.^23, 24, 25, 33^ Thus, miR-26 may also be involved in the development and progression of these tumors through the modulation of autophagy, and miR-26 overexpression may also sensitize these tumors to chemotherapeutic agent-induced apoptosis by inhibiting autophagy.

Although the dysfunction of miR-26 have been well documented in carcinogenesis and in the progression of various types of malignances including HCC.^25, 34^ However, the mechanism of the decreased expression of miR-26 is not clear. Here, using autophagy activators and inhibitors, we demonstrate that modulating autophagy directly affects the expression levels of miR-26a/b. 3-MA and the lysosome–autophagosome fusion inhibitor CQ increased the levels of miR-26a and miR-26b, whereas rapamycin decreased the levels of miR-26a and miR-26b, indicating that autophagy may promote the degradation of miR-26a and miR-26b in HepG2 cells. However, whether or how miR-26a/b is preferentially recruited to the autophagosome for lysosomal degradation remains to be further elucidated.

Autophagy acts as a double-edged sword in cancer. At the beginning of the tumor formation, autophagy is thought to act as a tumor suppressor by clearing mutant or misfolded proteins and alleviating cellular stress.^4, 35^ However, when a tumor is established, autophagy enables tumor cells to survive under nutrient deficiency, hypoxia and other stresses, or to form a barrier to treatment with chemotherapeutic drugs.^4^ Therefore, the inhibition of autophagy may benefit cancer treatment. However, most studies have focused on using general autophagy inhibitors, and most targets that have been screened for use in designing inhibitors are far downstream of the autophagy process.^8, 10^ Accumulating evidence suggests that the ULK1 is an attractive target for inhibiting autophagy.^29, 35, 36, 37^ As ULK1 is a key initiator of autophagy, Chan *et al.*^38^ used siRNA to inhibit the expression of ULK1. Egan *et al.*^29^ developed a small molecule inhibitor of ULK1 that acted synergistically with mTOR inhibition to enhance apoptosis in tumor cells. By overexpressing miR-26a/b *in vitro* and *in vivo*, we identified ULK1 as the common target of miR-26a/b; we also demonstrated the ability of miR-26/b to inhibit autophagy and sensitize HepG2 cells or implanted tumors to Dox treatment by suppressing ULK1 and downstream events, thereby revealing the therapeutic potential of miR-26a/b for use in combination with chemotherapy (Figure 7e).

In summary, by studying cell lines, clinical samples and mouse models, we have newly identified a regulatory mechanism of autophagy at the initiating stage and a novel biological function of miR-26a/b; these findings provide a novel cancer therapy strategy that combines the use of miR-26a/b with chemotherapy.

## Materials and methods

### Cells, animals and human tissues

HepG2, Huh-7, 293T cells were obtained from Shanghai Institute of Cell Biology, Chinese Academy of Sciences (Shanghai, China) and maintained in DMEM (Gibco, Carlsbad, CA, USA) supplemented with 10% fetal bovine serum and 1% penicillin–streptomycin within a humidified atmosphere containing 5% CO_2_ at 37 °C. The Dox-resistant HepG2 cells (HepG2/Dox) was established by continuous culture in medium containing stepwise increasing concentration of DOX at a range of 0.5–25 *μ*M over a period of 10 months. Cells using for functional and mechanism studies in this study were tested and authenticated using short tandem repeat method by Shanghai Institute of Cell Biology. HCC samples (*n*=30) were obtained from consenting patients, and experiments were approved by the Medical Ethics Committee of Gulou Hospital of Nanjing University. The clinical features of patients are listed in Table 1. The 9-week-old male SCID (severe combined immune deficiency) mice (nu/nu) were obtained from the Model Animal Research Center of Nanjing University (Nanjing, China) and maintained under specific pathogen-free conditions at Nanjing University.

### Transfection

MiRNA mimics, inhibitors and negative controls were purchased from Ruibo Company (Guangzhou, China). ULK1 small interfering RNA (sequence: 5′-GGUUAGCCCUGCCUGAAUCTT-3′) were purchased from GenePharma (Shanghai, China). For the ULK1 overexpression assay, a pcDNA3.1 vector was designed to specifically express the open reading frame of human ULK1 containing full-length 3′-UTR and purchased from GeneCopoeia (Germantown, MD, USA). Lipofectamine 2000 (Invitrogen, Carlsbad, CA, USA) was used for transfection according to the instruction. Rapamycin (200 nM), 3-MA (10 mM) or chloroquine (CQ, 50 *μ*M) (Sigma Aldrich, St Louis, MO, USA) and were added 18 h after transfection, if necessary.

### Western blotting

Cellular protein were extracted as described previously.^32^ Antibodies against ULK1, LC3 (Abcam, Cambridge, UK), Beclin-1 and ATG7 (Cell Signaling Technology, Beverly, MA, USA) were used for blotting. GAPDH (Santa Cruz Biotechnology, Inc., Santa Cruz, CA, USA) served as an internal control.

### RNA isolation and real-time quantitative PCR

Total RNA extraction, reverse transcription and TaqMan real-time polymerase chain reaction (PCR) for miRNAs were performed as described previously.^32^ Real-time PCR for mRNA detection were performed using SYBR Green PCR Master Mix (Ambion, Carlsbad, CA, USA). The sequences of the primers used were as follows: ULK1 mRNA (sense): 5′-CAGCAAAGGCATCATCCAC-3′, ULK1 mRNA (antisense): 5′-GGTTGCGTTGCAGTAGGG-3′, GAPDH (sense): 5′-GATATTGTTGCCATCAATGAC-3′ and GAPDH (antisense): 5′-TTGATTTTGGAGGGATCTCG-3′.

### Luciferase reporter assay

The 3′-UTR of human ULK1 containing putative binding sites was cloned into the p-MIR-REPORT plasmid (Ambion), and efficient insertion was confirmed by sequencing. To test the binding specificity, the sequences in human ULK1 3′-UTR that interact with miR-26a/b seed sequence were mutated (Mut-1, the first putative binding site from 5′-TACTTGAA-3′ to 5′-ATGAACTT-3′ Mut-2, the second putative binding site from 5′-TACTTGAA-3′ to 5′-ATGAACTT-3′ Mut-3, both putative binding sites from 5′-TACTTGAA-3′ to 5′-ATGAACTT-3′). 293T cells were co-transfected with *β*-galactosidase (*β*-gal) expression plasmid (Ambion), a firefly luciferase reporter plasmid, and miR-26a/b mimics or negative control. The *β*-gal plasmid was used as a transfection control. Luciferase activity was measured 24 h after transfection using a luciferase assay kit (Promega, Madison, WI, USA).

### Assessment of cell viability, apoptosis and the sensitivity of HCC cells to chemotherapy

Cell apoptosis under various treatments and cell viability was analyzed as described previously.^32, 39^ To analyze their sensitivity to chemotherapy, HepG2 cells transfected with miR-26a/b mimics or negative control were treated with Dox (range, 0–80 *μ*M) and cell inhibition was assessed by CCK-8 assay. The half-maximal inhibitory concentration 200 (IC50) was calculated.^39^

### Immunofluorescence, immunohistochemistry staining and electron microscopy

Immunofluorescent staining was done as described previously.^8, 10, 40^ In brief, tumor or liver slices were fixed and stained with primary, secondary antibodies prior to DAPI nuclear staining and mounted on to slides. Images were recorded using a Nikon microscope. GFP-LC3 puncta formation in HepG2 under the different treatments were determined by capturing images using a Nikon confocal microscope (Nikon, Tokyo, Japan) equipped with a × 100 oil immersion lens. The average number of GFP-LC3 dots per cell was calculated from at least 200 cells. To evaluate liver histological changes, liver sections were processed for H&E staining as described. Immunohistochemical staining of the paraffin sections was performed using a microwave-based antigen retrieval technique, specimen slides were incubated overnight at 4 °C with primary antibodies that were raised against Ki67 (Cell Signaling Technology). For electron microscopy, cells were treated as described previously and imaged on a JOEL JEM-1100 transmission electron microscopy (JEOL, Japan, Tokyo).^8^

### Animal model

HepG2 cells (1 × 10^6^ cells per mouse) were slowly injected into the livers of the mice. Three weeks later, the mice were divided into four groups of eight mice each. The groups of mice received PBS (100 *μ*l), PBS containing Dox (5 mg/kg), ~10^11^ titer/ml lentivirus-expressing miR-26 (LV-miR-26a/b) or PBS containing Dox and ~10^11^ titer/ml lentivirus-expressing miR-26 (LV-miR-26a/b). PBS, Dox and miR-26a/b-overexpressing lentiviral vector were administered by tail vein injection. PBS and Dox were injected every 3 days. The mice were killed 2 weeks later, and the tumors were dissected out. In a separate set of experiments, tumor-implanted mice were subjected to survival analysis.

### Statistical analysis

Each experiment is representative of at least three independent experiments. The data are presented as the means±S.E.M. of at least three independent experiments. Differences between groups were analyzed using Student's *t*-test. Differences between more than two groups were analyzed using ANOVA. Statistically significance was defined: *P<0.05; **P<0.01; and ***P<0.001.

## Figures and Tables

**Table 1:** Clinical features of the studied patients with hepatocellular carcinoma

**Number**	**Age**	**Gender**	**Pathological stage**	**HBV infection**
Case #1	66	Male	II	HBV+(004)
Case #2	74	Male	II	HBV+(005)
Case #3	56	Male	III	HBV+(93.57)
Case #4	61	Male	II	HBV+(>225)
Case #5	55	Female	I–II	HBV+(31.83)
Case #6	50	Male	II	HBV+(>225)
Case #7	50	Male	I–II	HBV+(25.723)
Case #8	48	Male	I	HBV+(>225)
Case #9	42	Male	II–III	HBV+(9.102)
Case #10	53	Male	III	HBV+(11.687)
Case #11	68	Male	II	−(0.001)
Case #12	48	Female	II	HBV+(72.519)
Case #13	54	Male	III	HBV+(>250)
Case #14	66	Male	II–III	HBV+(9.102)
Case #15	58	Male	—	HBV+(222.664)
Case #16	49	Male	—	HBV+(2.773)
Case #17	43	Male	III	HBV+(11.687)
Case #18	49	Male	II	−(0.001)
Case #19	55	Male	I	−(0.001)
Case #20	49	Male	—	HBV+(>225)
Case #21	63	Male	II	HBV+(72.519)
Case #22	64	Male	II	−(0.001)
Case #23	66	Male	III	HBV+(152.283)
Case #24	40	Male	III	HBV+(>250)
Case #25	45	Male	III	HBV+(>250)
Case #26	60	Female	I	HBV+(250)
Case #27	46	Female	II	−(0.001)
Case #28	41	Male	III	−(0.001)
Case #29	61	Male	I	HBV+(133.69)
Case #30	74	Male	I	−(0.001)

Abbreviation: HBV, hepatitis B virus

Pathological stage was classified according to Edmondson–Steiner^41^
