# Supplementary material for: MiR-26 enhances chemosensitivity and promotes apoptosis of hepatocellular carcinoma cells through inhibiting autophagy
Source: Cell Death Dis. 2017 Jan 12;8(1):e2540–. doi: 10.1038/cddis.2016.461 (PMC5386370; doi:10.1038/cddis.2016.461)
Supplement: Supplementary Information [file cddis2016461x1.docx]

**Supplementary Data to:**

**MiR-26 enhances chemosensitivity and promotes apoptosis of hepatocellular carcinoma cells through**

**inhibiting autophagy**

Fangfang Jin^1*^, Yanbo Wang^1*^, Mingzhen Li^1^, Yanan Zhu^1^, Hongwei Liang^1^, Chen Wang^1^, Feng Wang^2^, Chen-Yu Zhang^1#^, Ke Zen^1#^, and Limin Li^1#^

^1^State Key Laboratory of Pharmaceutical Biotechnology, Nanjing University Advanced Institute of Life Sciences, Jiangsu Engineering Research Center for MicroRNA Biology and Biotechnology, Nanjing University, Nanjing, Jiangsu 210093. ^2^Affiliated Gulou Hospital, Medical College of Nanjing University, Nanjing, Jiangsu 210093.

**Table of content**

[Supplementary Figure 1. Levels of LC3-I and LC3-II in HepG2 cells under Sorafenib treatment. 3](#_Toc456197085)

[Supplementary Figure 2. Levels of miR-26a/b in HepG2/Dox cells transfected with or without miR-26a/b mimics under different treatments. 3](#_Toc456197085)

[Supplementary Figure 3. Transfection of miR-26 a/b mimics or inhibitors can effectively upregulate or downregulate the expression of miR-26 a/b in Huh-7 and HepG2 cells. 4](#_Toc456197086)

[Supplementary Figure 4. Transfection of ULK1 expressing plasmids or ULK1 siRNAs can effectively upregulate or downregulate the expression of ULK1 in HepG2 cells 5](#_Toc456197087)

[Supplementary Figure 5. MiR-26a/b enhance the apoptosis of Huh-7 cells to chemotherapeutic drugs 6](#_Toc456197088)

[Supplementary Figure 6. MiR-26a/b enhances the sensitivity of HCC cells to sorafenib by inhibiting autophagy in vitro. 7](#_Toc456197089)

[Supplementary Figure 7. Stably expressing miR-26a/b in HCC cells inhibits autophagy, viability, apoptosis and tumor formation 8](#_Toc456197085)


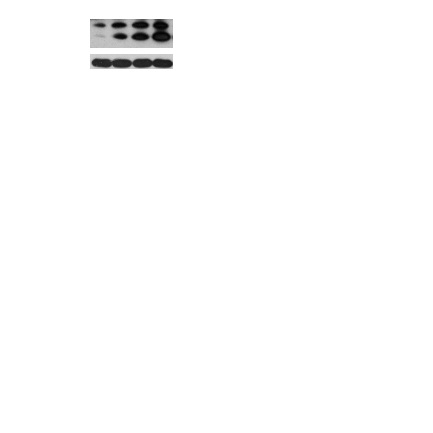

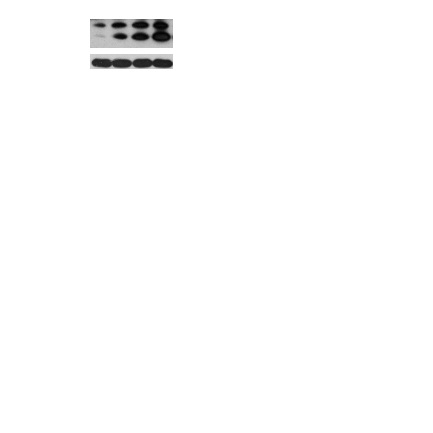


**GAPDH**

**LC3-II**

**LC3-I**

**DMSO**

**SORA**

**24h**

**48h**

**DMSO**

**SORA**

**Supplementary Figure 1. Levels of LC3-I and LC3-II in HepG2 cells under Sorafenib treatment.** Representative Western blotting analyses of LC3-I and LC3-II in HepG2 cells after treatment with Sorafenib (SORA) for 24 h and 48 h. The lower histograms represent quantitative analyses of LC3II/LC3-I protein levels.*** P < 0.001.

**
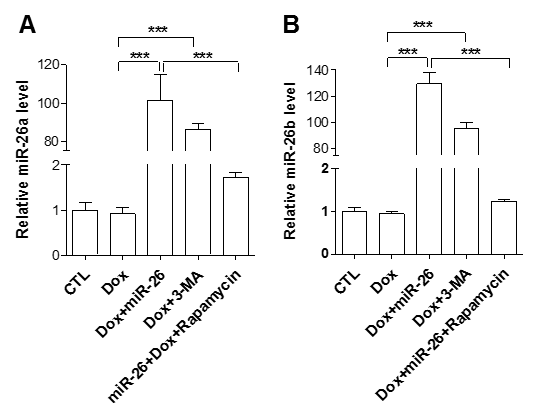
**

**Supplementary Figure 2. Levels of miR-26a/b in HepG2/Dox cells transfected with or without miR-26a/b mimics under different treatments.** HepG2 cells transfected with or without miR-26a /b mimics were treated with doxorubicin (Dox), 3-MA and/or rapamycin. MiR-26a (A) and miR-26b (B) levels were detected 24 h later respectively. *** P < 0.001.


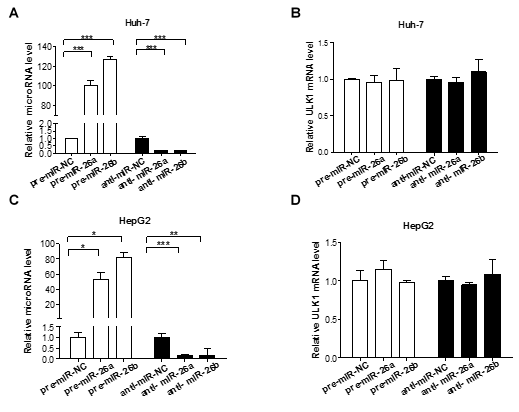


**Supplementary Figure 3. Transfection of miR-26a/b mimics or inhibitors can effectively upregulate or downregulate the expression of miR-26a/b in Huh-7 and HepG2 cells.** Huh-7 and HepG2 cells were transfected with miR-26a mimics (pre-miR-26a), miR-26b mimics (pre-miR-26b), random oligonucleotides of miRNA mimics (pre-miR-NC), random oligonucleotides of miRNA inhibitors (anti-miR-NC), miR-26a inhibitors (anti-miR-26a) and miR-26b inhibitors (anti-miR-26b) respectively. **(A and C)** Quantitative analysis of the levels of miR-26a and miR-26b by RT-qPCR in Huh-7 and HepG2 cells with different transfection. **(B and D)** Quantitative analysis of the levels of ULK 1 mRNA by RT-qPCR in Huh-7 and HepG2 with different transfection. All data are shown as mean ±S.E from three separate experiments.* P < 0.05; ** P < 0.01; *** P < 0.001.


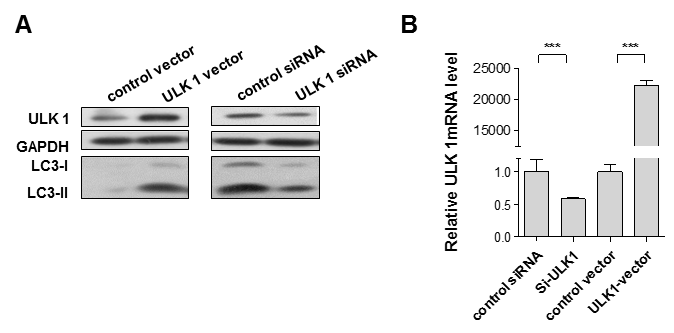


**Supplementary Figure 4. Transfection of ULK1 expressing plasmids or ULK1 siRNAs can effectively upregulate or downregulate the expression of ULK1 in HepG2 cells.** HepG2 cells were transfected with empty vector (control vector), ULK1 expressing plasmids (ULK1-vector), random siRNA oligonucleotides (control siRNA) or siRNA of ULK1 (ULK1 siRNA). **(A)** Protein levels of ULK 1 and LC3 were determined by western blotting in HepG2 cells with different transfection. GAPDH was served as internal control. **(B)** The level of ULK1 mRNA was detected by RT-qPCR in HepG2 cells with different treatment. Data are shown as mean ±S.E from three separate experiments. *** P < 0.001.

**Supplementary Figure 5. MiR-26a/b enhance the apoptosis of Huh-7 cells to chemotherapeutic drugs.** Huh-7 cells transfected with pre-miR-NC, pre-miR-26, ULK1-vector or pre-miR-26 plus ULK1-vector (pre-miR-26 + ULK1-vector). After transfection, cells were treated with or without Dox. Cell apoptosis of HepG2 cells under various treatments was analyzed using flow cytometry. Data are shown as mean ±S.E from three separate experiments. ** P < 0.01; *** P < 0.001.

**Supplementary Figure 6. MiR-26a/b enhances the sensitivity of HCC cells to sorafenib by inhibiting autophagy *in vitro*.**The sensitivities of HepG2 cells under different transfections with sorafenib were determined using a CCK-8 assay.


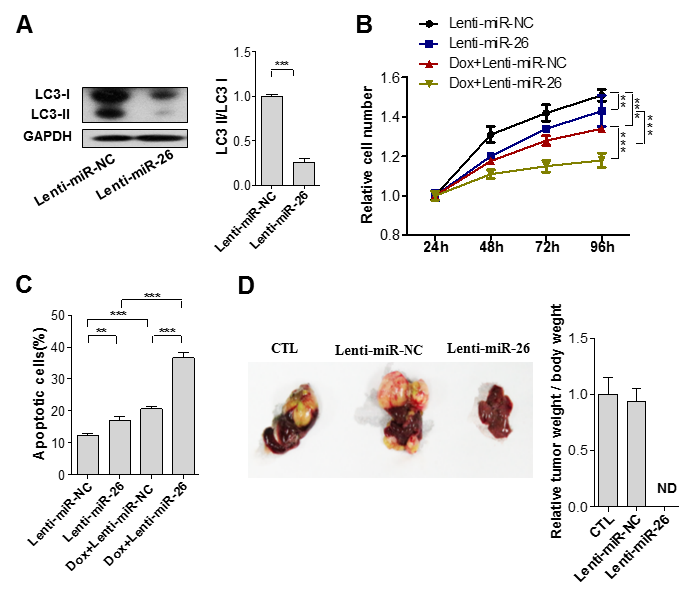


**Supplementary Figure 7. Stably expressing miR-26a/b in HCC cells inhibits autophagy, viability, apoptosis and tumor formation.** HepG2 cells stably expressing miR-26a and miR-26b were constructed using lentivirus-packaged miR-26a/b (Lenti-miR-26), and lentivirus-packaged empty vector (Lenti-miR-NC) was used as the control. **(A)** Protein levels of LC3-I and LC3-II in HepG2 cells expressing Lenti-miR-NC or Lenti-miR-26. The right histogram represents a quantitative analysis of ULK1 and LC3II/LC3-I protein levels. Cell viability **(B)** and apoptosis **(C)** of HepG2 cells expressing Lenti-miR-NC or Lenti-miR-26 with or without Dox treatment. **(D)** The same amounts of HepG2 cells without treatment (CTL), stably expressing Lenti-miR-26 or Lenti-miR-NC, were implanted into the livers of nude mice. The livers were collected 6 weeks after implantation. Statistical data are presented as the means ±S.E. from three independent experiments. ** P < 0.01; *** P < 0.001.
